# Oncologic surveillance for subjects with biallelic mismatch repair gene mutations-10 year follow-up in a kindred

**DOI:** 10.1186/1897-4287-9-S1-P11

**Published:** 2011-03-10

**Authors:** Carol A Durno, Melyssa Aronson, Uri Tabori, David Malkin, Helen Chan, Steven Gallinger

**Affiliations:** 1 Zane Cohen Familial Gastrointestinal Cancer Registry (FGICR) and Department of Surgery, Mount Sinai Hospital, Ney York, NY, USA; 2 Department of Pediatrics, Hospital for Sick Children, University of Toronto, Toronto, Ontario, Canada; 3 Division of Gastroenterology, Hepatology and Nutrition, Hospital for Sick Children, University of Toronto, Toronto, Ontario, Canada; 4 Division of Hematology/Oncology, Hospital for Sick Children, University of Toronto, Toronto, Ontario, Canada

## Background

Lynch syndrome (LS) is caused by heterozygous germline mutations in the DNA mismatch repair (MMR) genes and is a highly penetrant autosomal dominant condition. A novel childhood cancer syndrome caused by biallelic germline MMR gene mutations and characterized by brain tumors, leukemias, gastrointestinal (GI) polyposis, GI cancer and café-au-lait spots (CALS) has been described. We reported the first biallelic kindred in which 2 of 3 siblings proven to have a homozygous germline *MLH1* mutation, developed early-onset GI cancer. In contrast to LS with clear GI screening and surveillance recommendations, there are no recommendations for surveillance of individuals with biallelic mutations and no literature describing the long term outcome.

## Aim

To prospectively describe long-term outcome of our two young patients with biallelic MMR mutations, and to develop a generic cancer screening protocol for other patients with biallelic MMR mutations.

## Methods

On the basis of the molecular results, the 2 surviving sisters and parents of a deceased child with metastatic duodenal cancer began a surveillance protocol based on our crude estimates of cancer risks and available cancer screening modalities.

## Results

During endoscopic screening the youngest sister developed colonic polyps with high grade dysplasia and underwent a subtotal colectomy with ileorectal anastomosis. At 11 years she developed polyps in the duodenum with low grade dysplasia which were excised endoscopically. At 13 years of age, surveillance MRI revealed a left parieto-occipital anaplastic astrocytoma enabling total resection. Endoscopy in the older sister who had a subtotal colectomy with ileorectal anastomosis at 9 years of age for metastatic colorectal cancer has low grade polyps in the duodenum, ileum and rectum which were excised endoscopically. Gynecological follow-up of both sisters with annual pelvic ultrasounds have shown functional ovarian cysts. All neoplastic lesions identified during surveillance were asymptomatic at diagnosis. The sisters are currently fifteen and seventeen years old with no evidence of disease 10 years after their brother’s diagnosis. The parents (43 and 44 years) have annual colonoscopy with no evidence of polyps or cancer. Gynecological screening for the mother has not revealed any abnormalities. Based on our experience to date and review of available literature, we have developed the screening guidelines shown in Table 1.

**Table 1:** Screening Guidelines

Cancer	Recommendations
Colon	Colonoscopy yearly*
Upper GI Tract and small bowel	EGD yearly* Video capsule endoscopy *
Brain+	MRI brain q 6 months
Leukemia+ Lymphoma+	CBC, DNA for T-cell and B-cell rearrangement, serum LDH q 3 to 6 months
Urinary tract	Abdominal ultrasound at birth or diagnosis

EGD, Esophagogastroduodenoscopy

*beginning at 3 years of age or at diagnosis with frequency determined by baseline findings

+if diagnosed prenatally brain, leukemia/lymphoma screening should commence at birth

## Conclusions

Biallelic carriers who participated in oncologic surveillance had presymptomatic neoplasms identified and treated. These siblings are alive with no evidence of disease at 10-year follow-up. Aggressive surveillance in biallelic MMR carriers is feasible, allows early detection and improves long-term survival.

